# *Moltkia coerulea* extracts alleviate caspase-3 activity via reducing oxidative stress in LPS-induced neurotoxicity in BV-2 cells

**DOI:** 10.55730/1300-0144.6117

**Published:** 2025-09-29

**Authors:** Aslı CAN AĞCA, Derya ALTAY, Hüdaverdi KUL, Aslı F. CEYLAN, Betül SEVER YILMAZ

**Affiliations:** 1 Department of Traditional, Complementary and Integrative Medicine, Institute of Public Health, Ankara Yıldırım Beyazıt University, Ankara, Turkiye; 2 Department of Medical Services and Techniques, Vocational School of Health Services, Ankara Yıldırım Beyazıt University, Ankara, Turkiye; 3 Department of Medical Pharmacology, Faculty of Medicine, Ankara Yıldırım Beyazıt University, Ankara, Turkiye; 4 Department of Pharmacognosy, Faculty of Pharmacy, Ankara University, Ankara, Turkiye

**Keywords:** *Moltkia coerulea*, neurotoxicity, phenol profile, caspase-3, antioxidant

## Abstract

**Background/aim:**

*Moltkia coerulea*, a medicinal plant traditionally used for its neuroprotective properties, contains diverse phenolic compounds. However, its mechanisms of action in neuroinflammation remain unclear. We hypothesized that extracts of *M*. *coerulea* may protect microglial cells from lipopolysaccharide (LPS)-induced oxidative stress and apoptosis by modulating reactive oxygen species (ROS) and caspase-3 activity.

**Materials and methods:**

Aqueous and methanol extracts (5–50 μg/mL) were tested in BV-2 microglial cells. Cell viability was assessed by MTT assay, while ROS generation and caspase-3 activity were measured using fluorescence-based kits. The effective concentration (10 μg/mL) was selected for mechanistic assays based on viability data and previous studies. The phenolic profile of the plant extracts was determined by liquid chromatography with tandem mass spectrometry (LC/MS/MS).

**Results:**

Both extracts improved cell survival, reduced ROS levels, and attenuated caspase-3 activation in LPS-treated BV-2 cells. LC/MS/MS analysis identified chlorogenic acid, rosmarinic acid, vanillin, and rutin as predominant in the methanolic extract, while chlorogenic acid and rutin were most abundant in the aqueous extract; all are associated with antioxidant and antiapoptotic potential.

**Conclusion:**

*M*. *coerulea* extracts alleviated LPS-induced apoptosis in BV-2 cells by reducing oxidative stress and caspase-3 activity. These findings suggest a potential neuroprotective role of phenolic compounds present in *M*. *coerulea* that may translate into beneficial effects in animal models and warrant further preclinical evaluation.

## 1. Introduction

The *Moltkia* genus (Boraginaceae) has 4 taxa (3 species and 1 hybrid) in Türkiye. *Moltkia coerulea* (Willd.) Lehm. is widely distributed in Türkiye and also native to Iran, Lebanon–Syria, and across the Transcaucasus^1^ [1]. Throughout history, people have used plants for food, medicine, cosmetics, and health-promoting agents. Today, plant-based medicine is classified as being part of traditional medicine systems. The World Health Organization reported that over 40% of pharmaceuticals have a natural origin and traditional medicine is the source of some innovative medications^2^.The flowers and roots of *M*. *coerulea* are reported to be used against diarrhea and abdominal pain in Turkish folk medicine [2]. However, its neuroprotective mechanisms are not fully understood.

Oxidative stress occurs when the balance between free radicals, especially reactive oxygen species (ROS) production, and antioxidant mechanisms is broken. This imbalance causes the breakdown of molecular components, leading to cellular aging and cell death. It is commonly accepted that the oxidation of macromolecules, including lipids, proteins, and nucleic acids, in response to oxidative stress is linked to the development and progression of chronic illnesses [3–5].

The use of antioxidants is preferable for preventing an imbalance between oxidation and antioxidants. Aside from their role in decreasing oxidative stress, antioxidants have a variety of functions, including control of inflammatory pathways, improved cell function, and interaction with apoptosis-related enzymes. Natural or synthetic antioxidants safeguard cells from free radical-induced injury. In recent years, the use of natural antioxidants in the food, cosmetic, and pharmaceutical industries has emerged as a viable alternative to synthetic antioxidants due to their low cost and minimum adverse effects on the human body [6,7].

Among the secondary metabolites found in medicinal plants, phenolic and flavonoid compounds play a key role in modulating oxidative stress and apoptosis [8]. Chlorogenic acid, rosmarinic acid, vanillin, and rutin—identified as major constituents in this study—have been widely reported to have health benefits. Chlorogenic acid has antioxidant and antiinflammatory activities that may protect against neuroinflammation [9]. Rosmarinic acid has been shown to suppress ROS generation and inhibit caspase activation in neuronal models [10]. Vanillin, beyond its role as a flavoring agent, has radical-scavenging properties and neuroprotective effects [11]. Rutin, a well-known flavonoid, reduces oxidative stress, improves mitochondrial function, and modulates apoptotic signaling pathways [12]. Together, these phytochemicals may contribute to the potential neuroprotective effects of *M*. *coerulea*.

Neuroinflammation and oxidative stress are central contributors to the development of neurodegenerative diseases such as Alzheimer’s and Parkinson’s disease and generally refers to the inflammatory response that occurs by releasing inflammatory mediators by immune system cells, including neurons, microglia, astrocytes, and macroglia within the central nervous system [13]. Restoring homeostasis is the primary goal of the neuroinflammatory response—a process that removes pathogens and cellular waste while promoting tissue repair [14]. An important component of the outer membrane of gram-negative bacteria (such as *Escherichia coli*) that causes a strong inflammatory response is lipopolysaccharide (LPS), also referred to as endotoxin. Cytokines such as IL-1, IL-6, and TNF-α cause an increase in the synthesis of inflammatory mediators such as chemokines and free radicals. NF-κB activation triggers microglia-mediated phagocytosis, which releases cytokines and activating chemicals necessary for the adaptive immune response to function. Because LPS activates numerous inflammatory pathways in microglia, it is widely used to generate neurotoxicity models [15]. A variety of naturally occurring phenolic compounds possess antioxidant and/or antiinflammatory activities that may be helpful to mitigate or regulate neurotoxicity [16]. Similarly, *Moltkia* species have been reported to be rich in phenolic compounds that are considered potential therapeutic agents for the prevention or treatment of oxidative stress-related disorders [17–19].

Given the importance of microglial activation in neuroinflammation, the present study focused on the ability of *M*. *coerulea* extracts to attenuate oxidative stress and apoptosis in BV-2 microglial cells exposed to LPS and to establish the phenolic compound profile of the extracts by liquid chromatography with tandem mass spectrometry (LC/MS/MS). We hypothesized that *M. coerulea* extracts exert neuroprotective effects by reducing ROS production and caspase-3 activity, thereby alleviating LPS-induced apoptosis in BV-2 microglial cells.

## 2. Materials and methods

### 2.1. Chemicals

High-glucose Dulbecco’s modified Eagle medium (DMEM) with L-glutamine and fetal bovine serum (FBS) were purchased from Capricorn Scientific GmbH (Ebsdorfergrund, Germany). Trypsin, penicillin–streptomycin, and phosphate buffered saline (Dulbecco’s PBS 1×) were purchased from Cegrogen Biotech GmbH (Stadtallendorf, Germany). Dimethyl sulfoxide (DMSO) was purchased from BioShop Canada Inc. (Burlington, Ontario, Canada). MTT (3-(4,5-dimethylthiazol-2-yl)2,5-diphenyl-tetrazolium bromide) and LPS were purchased from Sigma-Aldrich (St. Louis, MO, USA). ROS detection and caspase-3 activity assay kits were purchased from Elabscience (Houston, TX, USA, catalog numbers: E-BC-K138-F and E-EL-R0160, respectively). Methanol, Folin–Ciocalteu, anhydrous sodium carbonate (Na_2_CO_3_), anhydrous aluminum chloride (AlCl_3_), anhydrous sodium acetate (NaOAc), gallic acid, quercetin, Trolox were purchased from Sigma-Aldrich (St. Louis, MO, USA), 2,2-diphenyl-1-picrylhydrazyl (DPPH) reagent was from Tokyo Chemical Industry (Tokyo, Japan) and 2,4,6-tris(2-pyridyl)-1,3,5-triazine (TPTZ) reagent were obtained from Sigma-Aldrich (St. Louis, MO, USA). Acetic acid, hydrochloric acid (HCl) and ferric chloride (FeCl_3_) heptahydrate were obtained from Merck (Darmstadt, Germany). Deionized (DI) water was used during this study.

### 2.2. Plant material

The flowering aerial parts of *M*. *coerulea* were collected from Ankara on 27 May 2021. The voucher specimen was deposited at the Herbarium of Ankara University Faculty of Pharmacy (AEF) with the number AEF30959. Professor Hayri Duman (Department of Biology, Faculty of Science, Gazi University, Ankara, Türkiye) approved the botanical authentication.

### 2.3. Preparation of extracts

The aerial parts of the plants were air-dried and powdered. Two separate extracts were prepared using methanol and water.

The plant materials used in this study were weighed accurately and extracted with methanol. Extraction was performed by using an ultrasonic bath (MIPRO MU C-28; Miprolab Lab. Cihazları A.Ş: Ankara, Türkiye) for 60 min at room temperature. The extracts obtained with methanol were evaporated until dry.

The air-dried and powdered plant materials were also weighed and extracted with water. Extraction was performed by using an ultrasonic bath (MIPRO /MU C-28) for 60 min at room temperature. The extracts obtained with water were lyophilized.

### 2.4. Analysis of total phenolic content

The total phenolic content of the plant extracts was determined according to the method of Singleton and Rossi [20]. In each well, 30 μL of the sample was mixed with 15 μL of Folin reagent, and left to incubate for 3 min in the dark. The wells were then filled with 5% Na_2_CO_3_ and distilled water, and incubated for 60 min. The absorbance value of the samples was obtained at 725 nm. The experiments were performed in triplicate. The same procedure was performed with gallic acid, and the results were expressed as milligrams of gallic acid equivalent (GAE) per gram of crude extract for each sample.

### 2.5. Analysis of total flavonoid content

The total flavonoid content of the plant extracts was determined according to the method of Zhishen et al. [21]. Briefly, plant extract samples, Na-acetate, and distilled water were added to each well. After 5 min of incubation, 10% aluminum chloride was added and samples were kept in the dark for 30 min. The absorbance was recorded at 425 nm. The tests were performed in triplicate. The same procedure was performed with quercetin and the results were expressed as milligrams of quercetin equivalent (QE) per gram of crude extract for each sample.

### 2.6. LC/MS/MS analysis

The phenolic content of water and methanolic *M*. *coerulea* extracts were determined and quantified using a Thermo Fisher Scientific (Waltham, MA, USA) Dionex Ultimate 3000 model UHPLC coupled with a tandem MS instrument (TSQ Quantum Access Max) and Xcalibur 2.2 software at the Hitit University Scientific Technical Application and Research Center [22]. The ionizations were detected using H-ESI. An ODS Hypersil (4.6 × 250 mm, 5 μm) column was used for chromatographic separation at 30 °C. For the mobile phase, a 0.1% formic acid solution in water (A) and methanol (B) was used in gradient elution. The gradient program was fixed as follows: 0–1 min, 0% B; 1–22 min, 95% B; 22–25 min, 95% B; 25–30 min, 100% B. The total evaluation time was 34 min with conditioning time. The solvent flow rate was 0.7 mL/min. The injection volume was 20 μL.

MS/MS parameters were set as follows: capillary temperature 300 °C, vaporizer temperature 350 °C, sheath gas pressure 30 Arb, auxiliary gas pressure 13 Arb, spray voltage (positive polarity) 4000, spray voltage (negative polarity) 2500, and discharge current 4.0 μA.

### 2.7. Cell culture studies

BV-2 microglial cells were cultured in high-glucose DMEM medium supplemented with 10% FBS, 1% glutamine, 1% penicillin–streptomycin, and 1% amphotericin B at 37 °C in an environment with 5% CO_2_. During the culturing period, the medium was changed at intervals of 2–3 days and checked under an inverted light microscope until the cells reached 70–80% confluency. Cells reaching sufficient confluency were passaged or replated.

### 2.8. Cell treatments

Upon reaching 70–80% confluency, BV-2 cells were seeded in 96-well culture dishes and incubated at 37 °C for 24 h in a 5% CO_2_ environment. The LPS-induced model, available elsewhere in the literature, was used to create the neuroinflammation model. Briefly, LPS (1 g/mL in PBS) was applied at increasing concentrations (1, 5, and 10 μg/mL) diluted in the medium for 24 h, followed by toxicity analyses. According to the concentrations and times defined, cell extracts and LPS were simultaneously applied to the cells to examine the therapeutic effect [23].

To evaluate the neuroprotective effects of *M*. *coerulea* extracts, BV-2 cells were divided into the following treatment groups:

**Control:** untreated cells.
**LPS only:** cells treated with 1 μg/mL LPS for 24 h.
**Extract + LPS:** cells treated with different concentrations of *M*. *coerulea* extracts (5–20 μg/mL) for 1, 6, and 12 h followed by LPS exposure for 24 h.

All treatments were performed in triplicate, and experiments were repeated 3 times independently.

### 2.9. Cell viability assay

Different concentrations (5–50 μg/mL) of extract were incubated for 1, 6, 12, and 24 h to evaluate early to late cellular responses.

The conventional MTT protocol—a colorimetric assay to monitor the metabolic activity of cells—was used for the analysis of cell viability. In brief, cells were seeded at 3 × 10^4^ cells/well in a 96-well culture dish and incubated with extracts or LPS alone or under established therapeutic effect protocols. After completing the incubation periods, the cells were maintained at 37 °C for 4 h with MTT solution (0.5 mg/mL) prepared in fresh medium. The medium was immediately removed, and the formazan crystals were dissolved in 100 μL DMSO. Absorbance values were measured at reference wavelengths of 490, 570, and 630 nm using a microplate reader (PowerWave XS2; BioTek Instruments, Inc., Winooski, VT, USA). The readings were expressed as a percentage of the control groups [23].

### 2.10. Reactive oxygen species assay

The concentration and time interval used in these experiments were obtained from the results of cell survival assays.

Intracellular ROS levels were measured using the 2′,7′-dichlorofluorescein diacetate (DCFH-DA) fluorescent probe [24]. After being cultivated in 96-well culture dishes at a concentration of 2 × 10^4^ cells/well, the cells were treated according to the methods established for colloidal platinum and LPS. After the incubation period, the cells were treated with 20 μM DCFH-DA in fresh media for 30 min at 37 °C. Esterases are responsible for the breakdown of DCFH-DA in cells, and the fluorescence of the oxidized dichlorofluorescein (DCF) molecule, which corresponds to the amount of ROS in the environment, could therefore be analyzed in a microplate reader at 488 nm excitation and 530 nm emission (Modulus microplate multimode reader, Turner BioSystems, Sunnyvale, CA, USA). To evaluate the effects of free radicals in the extracts, ROS formation was analyzed. A calibration curve was constructed by applying increased concentrations of H_2_O_2_ (50–1000 μM) to the cells for 12 h. The effects of both extracts in the presence of LPS on ROS levels were calculated according to the calibration curve.

### 2.11. Caspase-3 activity assay

The concentration and time interval used in these experiments were obtained from the results of cell viability assays.

A commercially available caspase-3 activity kit was used according to the manufacturer’s instructions for the studies. Briefly, each well in a 96-well plate contained 30 μL of cell lysate, 70 μL of assay buffer (50 mmol/L HEPES, pH 7.4, 0.1% CHAPS, 100 mmol/L NaCl, 10 mmol/L DTT, and 1 mmol/L EDTA), and 20 μL of caspase-3 colorimetric substrate Ac-DVD-pN. The 96-well plate was incubated at 37 °C for 2 h, allowing caspase in the sample to cleave the chromophore p-N from the substrate molecule. Absorbance values were collected at 405 nm, with the caspase-3 activity directly proportional to the colorimetric reaction [25].

### 2.12. Statistical analysis

All data are expressed as the mean ± SEM of 4–6 independent experiments. Statistical analysis was performed using 1-way ANOVA followed by Tukey’s posthoc test for multiple comparisons. A p-value less than 0.05 was considered statistically significant. Minimum degrees of freedom were maintained by ensuring at least 3 independent replicates per treatment.

## 3. Results

### 3.1. Phytochemical composition of *M. coerulea extracts*

The extract yields from methanolic and water extracts of *M*. *coerulea* aerial parts were 6.35% and 14.03%, respectively.

The total phenolic contents of the extracts were derived from the calibration curve of gallic acid (y = 0.083x − 0.0778, R^2^ = 0.9987). Additionally, the total flavonoid concentration of extracts was calculated using the quercetin calibration curve (y = 0.084x − 0.0939, R^2^ = 0.9954).

The total phenolic concentrations of the methanolic and aqueous extracts were similar (27.844 and 28.839 mg GAE/g, respectively). The methanolic extract had more than double the flavonoid content of the aqueous extract (23.628 and 10.975 mg QE/g, respectively) (Table 1). Moreover, qualitative and quantitative analyses were performed to screen phenolic compounds in both extracts by LC/MS/MS. Except for ellagic acid and resveratrol, the qualitative profiles of aqueous and methanol extracts were similar; however, the concentrations of the detected components differed. Chlorogenic acid (9805.167 μg/g), rosmarinic acid (2415.467 μg/g), vanillin (1813.357 μg/g) and rutin (1203.052 μg/g) were the most abundant compounds in the methanolic extract, while the major components in the water extract were chlorogenic acid and rutin (1148.833 and 1050.822 μg/g, respectively) (Table 2).

### 3.2. Effects of the extracts on BV‐2 cell viability

**Figure 1:**
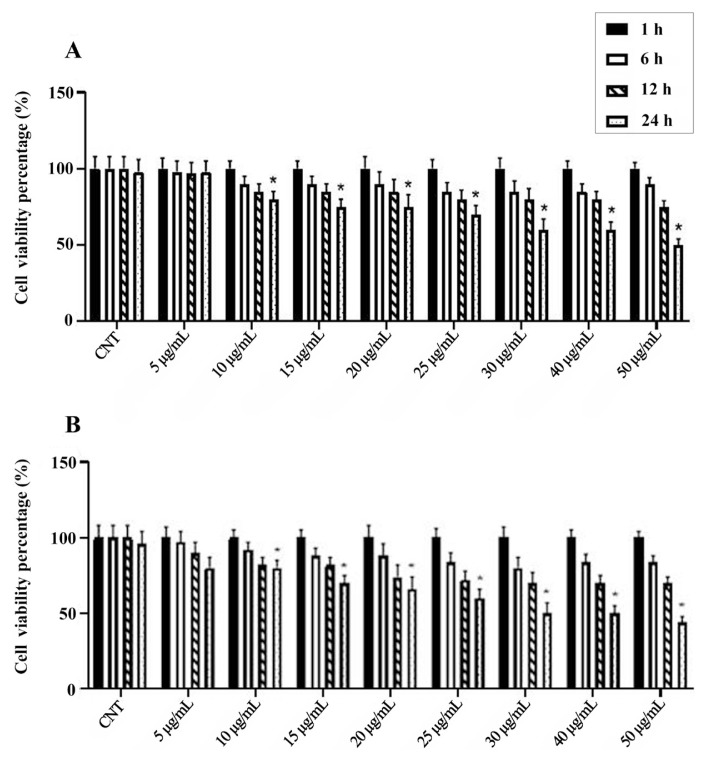
Effects of increasing concentrations of aqueous (**A**) and methanolic (**B**) extracts for 1, 6, 12, and 24 h on BV-2 cell viability. All data (n = 4) are expressed as the mean ± SEM. * = p < 0.05 vs. control counterparts.

The cytotoxicity of *M*. *coerulea* extracts was assessed using the MTT assay at concentrations of 5–50 μg/mL for 1, 6, 12, and 24 h. Cell viability was slightly reduced at higher concentrations (25 and 50 μg/mL, Figures 1A and 1B).

Based on the cell viability results, the concentration range of 5–20 μg/mL was used for mechanistic assays in the presence of LPS (1 μg/mL).

### 3.3. Extracts attenuate LPS-induced ROS production

**Figure 2:**
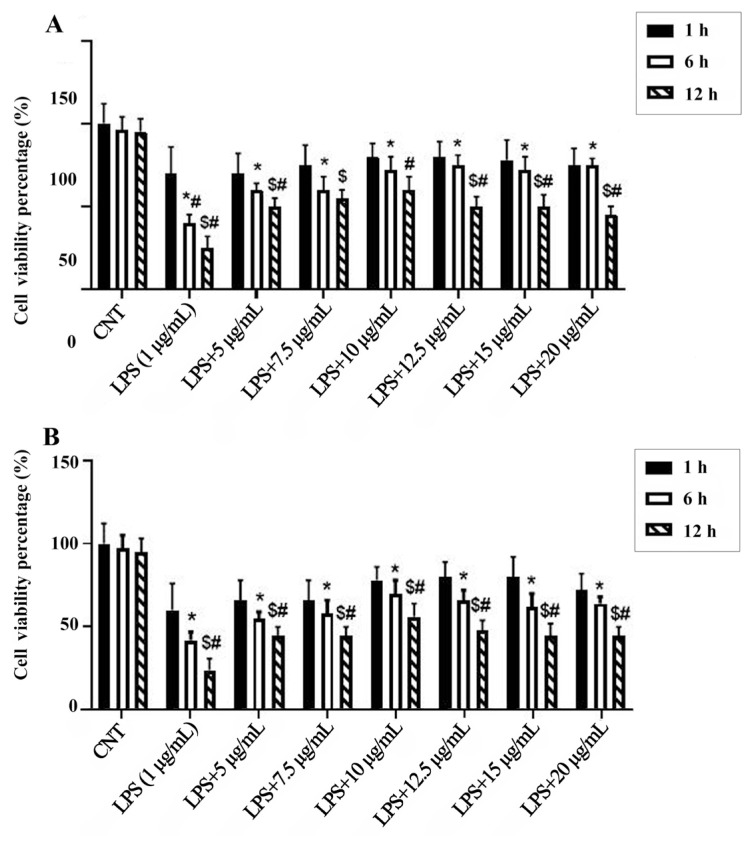
Effects of increasing concentrations of aqueous (**A**) and methanolic (**B**) extracts for 1, 6, and 12 h on BV-2 cell viability in the presence of LPS. All data (n = 4) are expressed as the mean ± SEM. * and $ = p < 0.05 vs. control counterparts and **#** = p < 0.05 vs. LPS (1 μg/mL at 1h).

**Figure 3:**
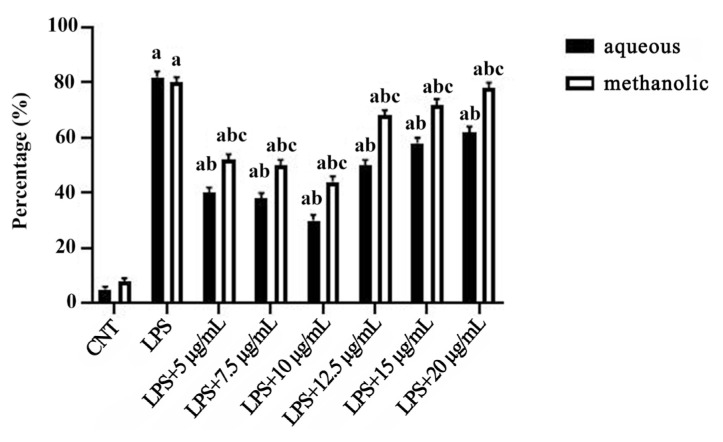
Effects of 12 h incubations of increasing concentrations of aqueous and methanolic extracts on ROS in the presence of LPS (1 μg/mL). All data (n = 6) are expressed as the mean ± SEM. **a** = p < 0.05 vs. control counterparts, **b** = p < 0.05 vs. LPS counterparts, and **c** = p < 0.05 vs. aqueous counterparts.

BV-2 cells exposed to LPS (1 μg/mL for 24 h) had a significant increase in intracellular ROS levels (approximately 2.5-fold vs. control, p < 0.001). Treatment with *M*. *coerulea* extracts significantly reduced ROS accumulation (approximately 1.3-fold vs. LPS alone, p < 0.05) (Figures 2A and 2B), and this reduction reached its maximum at 10 μg/mL (Figure 3).

### 3.4. Extracts suppress LPS-induced caspase-3 activation

**Figure 4:**
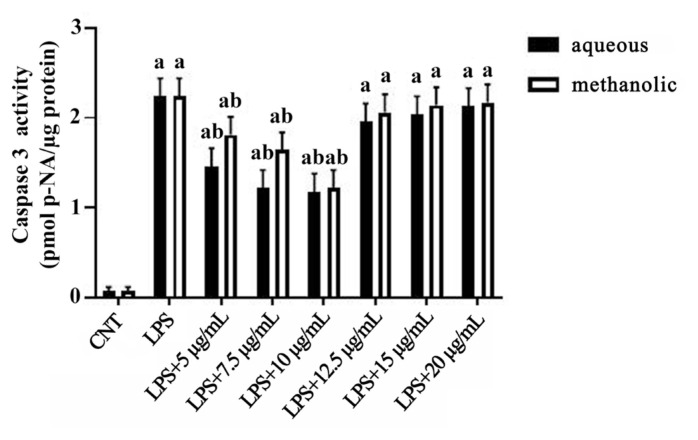
Effects of 12 h incubations of increasing concentrations of aqueous and methanolic extracts on caspase-3 activity in the presence of LPS (1 μg/mL). All data (n = 6) are expressed as the mean ± SEM. **a** = p < 0.05 vs. control counterparts and **b** = p < 0.05 vs. LPS counterparts.

Caspase-3 activity, a marker of apoptosis, was significantly elevated in LPS-treated BV-2 cells (approximately 3.0-fold vs. control, p < 0.001). Treatment with *M*. *coerulea* extracts markedly reduced caspase-3 activity (approximately 1.5-fold vs. LPS, p < 0.05) (Figure 4), and this reduction reached its maximum at 10 μg/mL.

## 4. Discussion

In this study, we screened the phenolic content of both aqueous and methanolic extracts of *M*. *coerulea* using LC/MS/MS and also investigated the effects of these extracts on LPS-induced neurotoxicity in the BV-2 cell model. Our results showed that *M*. *coerulea* extracts exert neuroprotective effects in LPS-induced BV-2 microglial cells by attenuating oxidative stress and reducing caspase-3 activity.

Phenolic compounds possess anticancer, antimicrobial, antiviral, antiangiogenic, antioxidant, neuroprotective, antitumor, and antiproliferative effects.

Additionally, recent studies have shown that chlorogenic acid, rosmarinic acid, vanillin, and rutin, which were detected as major compounds in *M*. *coerulea* in the present study, have multispectrum pharmacological benefits for the management of many chronic illnesses, including diabetes, cancer, high blood pressure, high cholesterol, and neurological disorders [26–29].

Zengin et al. [17] observed that protocatechuic acid, rutin, and hesperidin were the major components in the methanol extract of the aerial parts of *M*. *coerulea* (512.97, 340, and 244.32 μg analyte/g extract, respectively). They did not detect chlorogenic acid or vanillin, which were major compounds of the methanol extract of *M*. *coerulea* in our study. Zengin et al. [17] did find rosmarinic acid at a value of 84 μg, which we detected at 2415 μg. Rutin was reported to be lower than that found in our investigation. Total phenolic and flavonoid content were reported as 33.79 mg GAE/g extract and 34.85 mg RE/g extract, respectively. In the present study, total phenol and flavonoid contents of the extracts were lower than those found in the Zengin et al. [17] investigation. Another study examined water, ethyl acetate, and methanol extracts of different parts (flower, root, and leaf) of the plant. The highest total phenol content (252 mg GAE/g extract) was detected in aqueous root extract. Total flavonoid content was found to be higher in ethyl acetate extracts at 127.46 mg, 73.06 mg, and 62.67 mg QE equivalent/g extract in leaf, root, and flowers, respectively. Rosmarinic acid was higher in *M*. *coerulea* roots than in leaves and flowers. The rutin concentration was 0.099%, which is lower than the 0.25% in our study [13]. Another study on *M*. *coerulea* from Batman, Türkiye reported methanolic extracts of leaves and flowers had a better DPPH scavenging activity (61.2% and 40.17%, respectively) than water extracts (25.1% and 20.4%, respectively) [30]. The differences observed between our results and previous studies may be attributed to multiple factors that influence the phytochemical composition of plants. Environmental variables such as temperature, light exposure, humidity, and altitude can modulate the biosynthesis and accumulation of secondary metabolites. Since the biological activities of plant extracts are closely linked to the type and quantity of these metabolites, variations in geographical origin, environmental conditions, and genetic diversity may ultimately lead to differences in their pharmacological potential [31].

Oxidative stress plays an important role in the etiopathology of neurological diseases, and neuroinflammation constitutes an important component of progressive degenerative loss, mediating the pathogenesis of neurodegenerative diseases [32,33]. When microglia are activated by stimuli like LPS and viral infections, oxygen-free radicals and other inflammatory mediators are released, which can be directly harmful to neurons [34]. Microglial cells must have strong antioxidant defense mechanisms to prevent oxidative damage because they are frequently exposed to high concentrations of ROS [35].

In our study, significantly reduced ROS levels were found in the two extracts; however, the methanolic extract had a greater ROS-reducing capacity. This may be due to the higher values of phenolic compounds than those in the water extract.

LPS activates ROS signaling in microglial cells, causing the expression of inflammatory genes and inducing apoptosis in a caspase-3-dependent manner. Caspase-3 inhibition may prevent neuronal death and boost neuroprotective activity. As a result, apoptosis inhibitors may be a promising treatment strategy in neurodegeneration [36]. Both methanolic and aqueous extracts reduced caspase-3 activity mostly because of the alleviation of oxidative stress. Our findings show, for the first time, that *M*. *coerulea* extracts prevented apoptosis via reducing oxidative stress.

Natural substances with antioxidant properties can scavenge excess ROS and reactive nitrogen species, potentially preventing and treating different diseases. Evaluating long-standing usage in light of scientific evidence and additional research is the key stage for integrating traditional knowledge into conventional treatment. Considering all of the findings from this investigation, the extracts successfully alleviated oxidative stress, which in turn reduced caspase-3 activity in LPS-induced neurotoxicity. A standardized *M. coerulea* extract could be a useful therapeutic alternative to modulate neurotoxicity due to its bioactive constituents, particularly phenolic compounds.

## 5. Conclusion

This study shows that *M*. *coerulea* extracts mitigate LPS-induced oxidative stress and apoptosis in microglial cells, largely attributable to their phenolic constituents. Beyond cellular neuroprotection, these findings highlight the broader relevance of phenolic compounds in supporting human health by curbing oxidative damage and programmed cell death.

Targeting phenolic-rich plant extracts such as *M*. *coerulea* may provide a promising avenue for developing nutraceuticals or adjuvant therapies for neurodegenerative diseases. Future studies should validate these effects in animal models, explore the pharmacokinetics of key phenolics, and assess synergistic interactions with existing neuroprotective agents.

## Figures and Tables

**Table 1:** Total phenolic and flavonoid contents of plant extracts.

	mg GAE/g extract		mg QE/g extract	
	Mean	SD	Mean	SD
Methanolic extracts	27.844	2.057	23.628	2.192
Aqueous extracts	28.839	2.965	10.975	0.370

**Table 2:** Phenolic compounds identified and quantified in aqueous and methanol extracts of *M*. *coerulea* (μg/g dry extract).

Compound	Rt	Parent ion (m/z)	Ion. mode	MS/MS (CE)	%RSD	Linearity range (ppm)	Linear regression equation	R^2^	LOD/LOQ mg/L	Aqueous extract	Methanolic extract
Gallic acid	9.19	169.7	Neg	80.50 (25)–126.20 (16)	4.5	0.125–10	y = −1772.12+66065.7X	0.9949	0.061/0.203	nd	nd
Protocatechuic acid	11.11	155.01	Pos	65.40 (22)–93.20 (13)	4.9	0.125–10	y = 27640.8+35692.1X	0.9926	0.049/0.162	310.333	701.741
Protocatechuic aldehyde	12.07	136.9	Neg	92.25 (25)–108.20 (25)	2.2	0.125–10	y = 506291+900489X	0.9963	0.026/0.087	216.980	152.550
Sesamol	12.07	137.18	Neg	108.17 (15)–109.29 (14)	2.1	0.125–10	y = 190429+294285X	0.9965	0.031/0.103	56.874	18.067
Gentisic acid	12.34	153.7	Neg	109.50 (21)	2.3	0.125–10	y = 81035.3X	0.9979	0.013/0.043	nd	nd
Catechin	12.58	289.2	Neg	203.90 (22)–245.70 (17)	2.5	0.125–10	y = −46484.8+91012.1X	0.9987	0.042/0.058	nd	nd
Chlorogenic acid	13.61	353.4	Neg	86.50 (43)–192.10 (21)	3.5	0.125–10	y = 21653.3X	0.9937	0.106/0.353	1148.833	9805.167
Epicatechin	13.99	291.5	Pos	123.30 (15)–139.30 (16)	2.5	0.125–10	y = −137604+217920X	0.9951	0.003/0.006	nd	nd
Caffeic acid	14.19	179.7	Neg	135.20 (27)–136.20 (18)	2.5	0.125–10	y = 166126+470479X	0.9981	0.042/0.058	53.935	1.406
Vanilin	14.71	150.91	Neg	92.30 (23)–136.10 (16)	3.7	0.125–10	y = 18407.3+4851.4X	0.9937	0.023/0.076	515.268	1813.357
Syringic acid	15.21	183.07	Neg	77.30 (23)–123.20 (13)	4.6	0.125–10	y = 150673–55037.7X	0.9149	0.194/0.647	nd	nd
Taxifolin	15.71	303	Neg	126.20 (23)–285.50 (15)	3.8	0.125–10	y = 8374.89+42570.1X	0.9979	0.001/0.005	nd	nd
p-Coumaric acid	15.86	163.9	Neg	94.30 (33)–120.20 (17)	3.5	0.125–10	y = 79931.9+73318.2X	0.9927	0.069/0.109	nd	nd
Ferulic Acid	16.13	195.022	Pos	89.40 (30)–177.40 (7)	4.5	0.125–10	y = 96528.3+177434X	0.9941	0.063/0.118	139.085	386.588
p-Hydroxybenzoic acid	16.37	137.9	Neg	66.60 (38)–94.60 (17)	4.7	0.125–10	y= 537544+881649X	0.9951	0.243/0.809	nd	nd
Salicylic acid	16.38	137.136	Neg	65.50 (35)–93.26 (18)	4.8	0.125–10	y= 485398+972318X	0.9960	0.030/0.099	nd	nd
Hesperidin	16.78	608.78	Neg	301.34 (32)–164.01 (60)	4.3	0.125–10	y= 1.43184e+006X	0.9977	0.104/0.347	45.351	5.413
Rosmarinic acid	16.86	359.18	Neg	134.30 (44)–162.20 (20)	2.6	0.125–10	y = −127182+253431X	0.9980	0.003/0.005	360.115	2415.467
Oleuropein	16.95	539.1	Neg	275.80 (22)–377.50 (16)	2.0	0.125–10	y= 375327+1.76005e+006X	0.9969	0.009/0.029	nd	nd
Luteolin-7-glucoside	17.30	446.89	Neg	284.00 (45)–285.00 (40)	4.1	0.125–10	y= 135610X	0.9985	0.117/0.390	nd	nd
Rutin	17.48	609.37	Neg	300.60 (38)–301.70 (34)	1.6	0.125–10	y=2.20056e+006+1.00456e+007X	0.9977	0.022/0.073	1050.822	1203.052
Resveratrol	17.59	228.98	Pos	107.20 (22)–135.10 (14)	4.8	0.125–10	y= 97778.9+63601.2X	0.9988	0.019/0.062	20.762	nd
Ellagic acid	18.61	301.669	Neg	284.80 (30)–174.15 (34)	5.0	0.125–10	y= 745549+1.07598e+006X	0.9977	0.087/0.289	11.279	nd
Naringenin	19.08	273	Pos	147.10 (20)–153.00 (24)	4.8	0.125–10	y= 2.35935e+006X	0.9986	0.005/0.017	nd	nd
Quercetin	19.75	301	Neg	152.10 (23)–179.90 (20)	2.9	0.125–10	y=111352+2.43427e+006X	0.9941	0.001/0.005	nd	nd
Luteolin	20.23	287.02	Pos	135.10 (32)–153.00 (31)	3.9	0.125-10	y = 1.48908e+007X	0.9981	0.222/0.741	nd	nd
Apigenin	21.15	268.86	Neg	117.19 (40)–149.11 (27)	3.9	0.125–10	y = 831953X	0.9987	0.114/0.380	43.675	368.451
Pinocembrin	21.19	257.07	Pos	153.11 (23)–103.27 (34)	1.4	0.125–10	y = 789059X	0.9979	0.095/0.316	nd	nd
Chrysin	22.59	254.97	Pos	153.10 (30)–103.28 (33)	1.5	0.125–10	y= 1.33937e+007X	0.9972	0.049/0.163	nd	nd
Galangin	22.64	268.85	Neg	169.15 (29)–171.08 (31)	5.0	0.125–10	y= 1.66971e+006X	0.9929	0.034/0.113	nd	nd
Flavone	22.94	222.9	Pos	77.28 (35)–121.15 (26)	1.7	0.125–10	y= 1.57469e+007+3.51252e+007X	0.9954	0.037/0.124	nd	nd

**Parent ion (m/z):** Molecular ions of the standard compounds, **MS/MS(CE):** collision energy, **R^2^**: coefficient of determination, **Rt:** retention Time, **LOD**: limit of detection, **LOQ:** limit of quantification, **RSD**: relative standard deviation, **Neg:** negative ionization mode, **Pos:** positive ionization mode.

## Footnotes

1 Plants of the World Online. *Moltkia coerulea* (Willd.) Lehm. [online]. Website https://powo.science.kew.org/taxon/urn:lsid:ipni.org:names:118802-1. [Accessed 15 November 2024]

2 World Health Organisation (2023). Traditional medicine has a long history of contributing to conventional medicine and continues to hold promise. [online]. Website https://www.who.int/news-room/feature-stories/detail/traditional-medicine-has-a-long-history-of-contributing-to-conventional-medicine-and-continues-to-hold-promise. [Accessed 11 November 2024] .
